# Up-Converting Nanoparticle-Based Immunochromatographic Strip for Multi-Residue Detection of Three Organophosphorus Pesticides in Food

**DOI:** 10.3389/fchem.2019.00018

**Published:** 2019-02-07

**Authors:** Rubing Zou, Yunyun Chang, Tianyi Zhang, Fangfang Si, Ying Liu, Ying Zhao, Yihua Liu, Mingzhou Zhang, Xiaoping Yu, Xusheng Qiao, Guonian Zhu, Yirong Guo

**Affiliations:** ^1^Institute of Pesticide and Environmental Toxicology, Ministry of Agriculture Key Laboratory of Molecular Biology of Crop Pathogens and Insects, Zhejiang University, Hangzhou, China; ^2^Research Institute of Subtropical Forestry, Chinese Academy of Forestry, Hangzhou, China; ^3^College of Life Sciences, China Jiliang University, Hangzhou, China; ^4^Department of Materials Science and Engineering, Zhejiang University, Hangzhou, China

**Keywords:** organophosphorus pesticide, up-conversion nanoparticles, lateral flow strip, immunoassay, multi-residue detection

## Abstract

Organophosphorus (OP) pesticides are widely used to control pests because of their high activity. This study described a rapid and sensitive lateral flow immunochromatographic (LFIC) assay based on up-converting nanoparticles (UCNPs) for multi-residue detection of three OP pesticides. The developed assay integrated novel fluorescent material UCNPs labeled with a broad-specific monoclonal antibody. Based on the competitive platform by immobilized antigen in the test zone, the optimized UCNPs-LFIC assay enabled sensitive detection for parathion, parathion-methyl, and fenitrothion with IC_50_ of 3.44, 3.98, and 12.49 ng/mL (*R*^2^ ≥ 0.9776) within 40 min. The detectable ability ranged from 0.98 to 250 ng/mL. There was no cross-reactivity with fenthion, phoxim, isocarbophos, chlorpyrifos, or triazophos, even at a high concentration of 500 ng/mL. Matrix interference from various agricultural products was also studied in food sample detection. In the spiked test, recoveries of the three OP pesticides ranged from 67 to 120% and relative standard deviations were below 19.54%. These results indicated that the proposed strip assay can be an alternative screening tool for rapid detection of the three OP pesticides in food samples.

## Introduction

Organophosphorus (OP) pesticides, a class of insecticides, are widely used in pest control for their strong effect and broad spectrum (Roberts and Aaron, 2008; Handford et al., 2015). As an inhibitor of acetylcholinesterase, OP pesticides cause the accumulation of neurotransmitters and dysfunctional neuropathy (Davies et al., 1996; Payne-sturges et al., 2009; Bouchard et al., 2011), which pose a significant threat to human health and food safety. Considering their high toxicology, many counties set strict regulations to defend against hazards from OP pesticides, as parathion and parathion-methyl were banned in the European Union, Australia and China. Unfortunately, the illegal use of them as hidden components still occurs in some areas. The potential risk of excessive residue in agriproducts is also highlighted (Liang et al., 2012). Therefore, there is an urgent need to establish highly sensitive analytical methods for large-scale monitoring of OP pesticide residue.

Many instrumental analysis methods have been established for the determination of OP pesticide residues. For example, gas chromatography (GC), high performance liquid chromatography (HPLC), and GC coupled with mass spectrometry (GC-MS) were widely used due to their high accuracy and sensitivity (Liang et al., 2008; Tao et al., 2013; Watanabe et al., 2014; Zhao et al., 2014). However, these methods are demand much in the way of equipment and professional operating technology. As an alternative, the immunoassay based on the specific combination of antigen and antibody has high sensitivity and specificity. Combining simple operation with economical material price, the immunoassay has gradually become an important detection method. However, enzyme-linked immunoassays still require laboratory operations such as multiple incubations, washing, and sample pretreatment steps, hindering application in fields and markets (Xu et al., 2011). There is high demand for developing a fast and on-site method for the determination of OP pesticides in food and environmental samples.

Lateral-flow immunochromatographic (LFIC) assay, an ideal one-step immunoassay technique, was introduced to a variety of areas for rapid detection outside the laboratory (Wang et al., 2009; Zhu et al., 2011) as a result of its great convenience. As label selection is vitally important to the assay sensitivity, colorimetric and fluorometric measurement are usually considered (Xie et al., 2014). Colloidal gold (CG) has been the most widely used label in LFIC strips because of its low-cost preparation and visual detection. However, due to the limited signal-to-noise ratio of visible lights, CG-LFIC assays may have the drawbacks of low sensitivity and a narrow detection range (Liu et al., 2012). Therefore, different fluorescence tracers are employed to LFIC assays, such as quantum dots (QDs) (Qi et al., 2016) and fluorescence microspheres (Huang et al., 2016).

Up-converting nanoparticles (UCNPs), a kind of novel fluorescence material with special luminescence properties, possess the unique optical ability to up-convert low-energy infrared radiation and emit high-energy visible light. In theory, they can circumvent the background fluorescence from biological samples. In addition, as an inorganic inert material, these particles can avoid fluorescence-quenching decay. Thus, UCNPs are beneficial for the improvement of sensitivity and accuracy with less influence from the sample matrix (Dou et al., 2013; Feng et al., 2013), and the UCNP-LFIC assays have shown great potential in the field of large-screening and point-of-care testing. According to the literature, UCNP-LFIC assays have been developed for the detection of *Vibrio anguillarum* (Zhao et al., 2014), foodborne pathogens (Zhao et al., 2016a), Neutrophil gelatinase-associated lipocalin (NGAL) (Lei et al., 2017), *Yersinia pestis* (Yan et al., 2006), and prognosis of heart failure (You et al., 2017). For the determination of small molecular compounds like pesticides, UCNP-LFIC assays require a competitive format and face a great challenge in detection. At present, only very few UCNP-LFIC assays have been established for the determination of small-molecular contaminants, such as aflatoxin B1 (Zhao et al., 2016b) and clenbuterol (Wang et al., 2016) as well as imidaclothiz (Hua et al., 2018).

In this study, we focused on preparing UCNP fluorescent probes coupled with a broad-specific monoclonal antibody (mAb) that can recognize parathion, methyl-parathion and fenitrothion simultaneously. A competitive UCNP-LFIC assay was further established for rapid quantitative determination of three OP pesticides with high sensitivity. Meanwhile, multiple detections for various agricultural matrix interference tolerance degrees were also reviewed, enabling it to screen the three OP pesticides in food samples within 40 min. To the best of our knowledge, this is the first report of using UCNP-LFIC assays for OP pesticide residue detection. It thus furthers the application of UCNP-LFIC assays in the field of food safety and quality monitoring.

## Materials and Methods

### Materials and Reagents

Carboxylic acid-functionalized UCP (NaYF_4_: Yb^3+^, Er^3+^) nanoparticles (diameter of 150 nm; excitation spectrum peak of 545 nm; emission spectrum peak of 660 nm) were obtained from Fluo Nanotech Co., Ltd (Hangzhou, China). 1-(3-Dimethylaminopropyl)-3-ethylcarbodiimide hydrochloride (EDC, 99%) was purchased from Sigma-Aldrich (St. Louis, MO, USA). N-Hydroxysulfosuccinimide sodium salt (sulfo-NHS, 99%), trahalose (99%), polyvinyl pyrrolidone and Tween-20 were purchased from Aladdin Industrial Corporation (Shanghai, China). 2-(N-morpholino) ethanesulfonic acid (MES) was supplied by Sangon Biotech Co., Ltd (Shanghai, China), bovine serum albumin (BSA) was obtained from Sino-American Biotechnology (Luoyang, China). N-propyl-ethylenediamine (primary secondary amine, PSA) was supplied by Agela Technologies (Tianjin, China).

Nitrocellulose membranes (MDI 90, 2.0 cm × 30 cm), glass fibers, plastic adhesive backing pad, and absorbent pad were obtained from Jiening Biotech CO., Ltd (Shanghai, China). Plastic card sets fit for the reading instruments were designed by our group.

A broad-specific mAb against parathion and its coating antigen PA0304-OVA were previously produced in our laboratory (Jiao et al., 2018). Goat anti-mouse IgG was obtained from Biodragon Immunotechnologies (Beijing, China). Standards of OP pesticides, including parathion, parathion-methyl, fenitrothion, fenthion, phoxim, isocarbophos, chlorpyrifos, and triazophos, were provided by the Agro-Environmental Protection Institute, Ministry of Agriculture (Tianjin, China). All reagents were of analytical grade, used without any purification.

### Apparatus

The size and surface morphology of UCNPs were characterized by transmission electron microscope (TEM, HITACHI, Japan). The F-4500 fluorescence spectrometer system (HITACHI, Japan) adapted with a 980 nm laser device (Changchun Laser Optoelectronics Technology, China) was used to determine the fluorescent spectrum. Immunoreagents were dispensed on nitrocellulose membrane by R5DDA dispense platform (Han'gan, China). Strips were prepared by a cutter (Han'gan, China). The UCNP-based LFIC (UCNP-LFIC) strips were scanned by a strip reader (Suzhou Helmen Precise Instruments, China) with 980 nm near-infrared laser excitation. An ML-902 magnetic stirring apparatus (Pujiang, Shanghai), Allegra 64R super centrifuge (Beckman, America), electric jacket and ultrasonic cleaner were also used in this study.

### Preparation of UCNP-mAb Probe

**Scheme 1:**
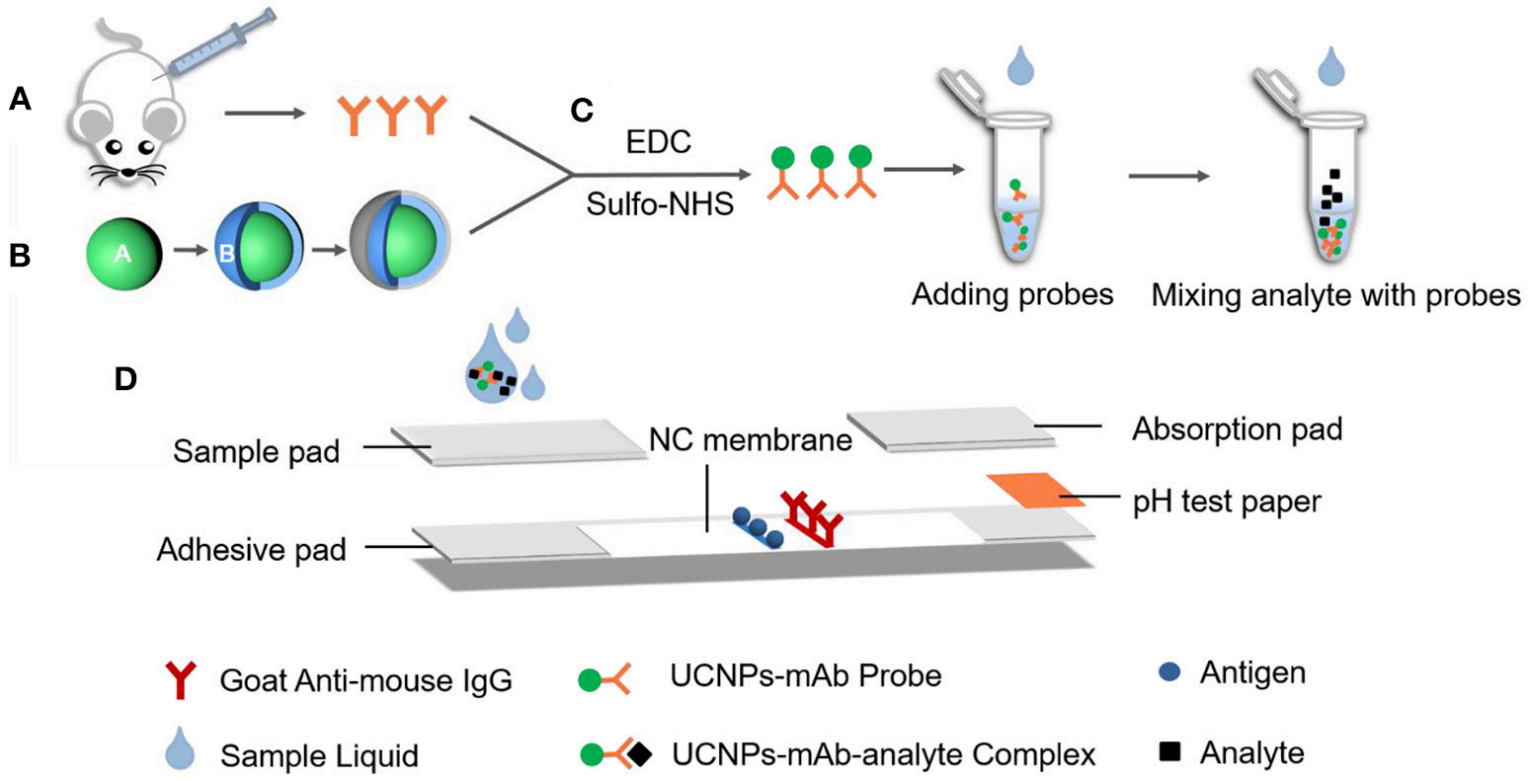
The fabrication process of lateral flow strips on UCNP-mAb probe. **(A)** MAb originated from BALB/c mice; **(B)** Carboxylic acid-functionalized UCNPs; **(C)** Bio-conjugation of functionalized UCNPs with mAb to obtain UCNPs-mAb; **(D)** Structure of later flow immunochromatographic strip.

To obtain fluorescent probe, the mAb originated from BALB/c mice (Scheme 1A) was conjugated with the functional UCNPs (Scheme 1B) via EDC/sulfo-NHS mediated amidation reaction (Scheme 1C), similar to the method described in our previous work (Si et al., 2018). The modified protocol is as follows: 1 mg of carboxylic UCNPs was dissolved in 2 mL MES (0.1 mol/L, pH 5.5) solution, then activated by adding 1 mg of EDC and 1.5 mg of sulfo-NHS. After 20 min of incubation with vigorous stirring at room temperature (RT), the activated product was centrifuged and washed two times with PB (0.01 mol/L, pH 7.4), and dissolved in 1 mL of PB. Then, 1 ml of mAb at concentrations of 10, 20, 40, and 80 μg/mL were added and stirred softly in RT for 2.5 h. Afterward, 250 μL of 1% BSA (w/v) was added as a blocking buffer to avoid non-specific locus for 30 min. Finally, after being washed two times, the precipitates were resuspended in 1 mL of the preservation solution (0.03 mol/L, pH 7.2 PB buffer containing 1% BSA, 1% trehalose, and 0.1% Tween-20) for storage. The resulting UCNP-mAb probe was stored at 4°C for further use.

### Preparation of Colloidal Gold-mAb Probe

According to the procedures used in our previous work (Guo et al., 2009), CG nanoparticles were prepared with an average diameter of 20 nm and coupled with the same specific mAb.

### Construction of Lateral Flow Strips

The fabrication of the lateral flow strips are as follows. Sample pad, nitrocellulose membrane, absorption pad, and pH test paper were stuck to a plastic adhesive backing pad, with 2 mm overlap between each two adjacent parts (Scheme 1D). Acting as a pump, the absorption pad was capable of liquid flow. Meanwhile, pH test paper also indicated the sample solution migrated properly through the strip. The goat anti-mouse IgG (0.3 mg/mL) and the competitive antigen of PA0304-OVA (3 mg/mL) were dispensed on the nitrocellulose membranes (0.75 μL/cm) and served as control and test lines with a 5 mm distance. Afterwards, the immobilized components were dried at 37°C for 30 min. The sample pad was pre-treated by blocking solutions, followed by drying at 37°C for 3 h. Different nitrocellulose membranes and blocking solutions were compared in this study. After overall assembly, the objective pad was cut into strips with a width of 4 mm and then stored in a sealed storage container for drying at RT for further use.

### Detection Procedure of UCNP-LFIC Strip

The UCNP-mAb probe at a specific amount was mixed with 60 μL of PBS buffer containing organic solvent, shaking gently to form the homogeneous sample solution. Subsequently, the mixture was dropwise added onto the sample pad. After 15 min, the color of the pH test paper turned red, indicating successful movement of mixture liquid on the strips. The fluorescence intensity values of the T (FI_T_) and C (FI_C_) lines were recorded.

### Quantitative Analysis of UCNP-LFIC Strip

By detecting a series concentration of standard analyte solution with a final concentration from 0 ng/mL (as negative for blank control) to 250 ng/mL (as positive), competitive inhibition curves of the three OP pesticides and their mixed solutions were established. The mixed standard solution was prepared by adding parathion, parathion-methyl, and fenitrothion together to the same final concentration. The ratio of FI_T_ to FI_C_ (FI_T_/ FI_C_) were calculated as B (positive sample) and B_0_ (negative control), respectively. A fitted standard curve was obtained for the inhibition rate (B_0_-B)/B_0_ × 100% against the series of concentration. The concentration causing 50% inhibition (IC_50_) was used to evaluate the assay sensitivity.

### Optimization of UCNP-LFIC Strip

According to the maximum fluorescence, signal-noise ratio and IC_50_, variable factors (antigen and antibody dilution times, the concentration of organic solvent in reaction buffer and detection time) were compared based on the sensitivity performance.

### Evaluation of the Assay Specificity

Eight types of OP pesticides were employed to determine the specificity of the assay. The pesticide standard solutions were diluted to a final concentration of 500 ng/mL as described above and tested individually.

### Sample Pretreatment and Spiked Recovery Test

Fruit and vegetable foods including orange, cucumber, and tomato were purchased from a Walmart supermarket. West Lake water, Yuhangtang River water, and tap water were obtained in Hangzhou, China. All samples were confirmed by GC/MS to be OP pesticide-free. The samples were pretreated with a simplified QuEChERS extraction procedure. Briefly, 10 g of homogenized sample was extracted with 20 mL of acetonitrile for 10 min by vigorous shaking, 1 g of NaCl and 5 g of MgSO_4_ were poured and shaken by hand for 2 min. After being centrifuged at 3,500 g for 5 min, 50 mg PSA was added and supernatants were treated by nitrogen and diluted with a reaction buffer for analysis. The water sample was filtered after standing for 5 min to remove solid impurities such as gravel and sand, and a certain amount of methanol was added to the liquid, leading to a final concentration of 10% before testing.

For the recovery test, each sample was spiked with different concentrations of OP pesticides and rested for 2 h before sample preparation, followed by testing in triplicate with the UCNP-LFIC assay.

## Results and Discussion

### Principle of the Detection Platform

For multiple target detection, the LFIC assay usually focused on the following two types of technician paths: (1) several channels providing independent reaction areas for single-target (Zhao et al., 2016a); (2) several test lines targeting different specific UCNP-mAb probes by utilizing mono-color or multi-color fluorescent nanoparticles (Liu et al., 2017). In this study, we used a broad-specific mAb (PA-QA1-7B2) for simultaneous multi-residue detection of parathion, parathion-methyl, and fenitrothion. The broad-specific mAb was produced and their similar binding properties against three OP pesticides were proved by surface plasmon resonance-based immunosensor-based immunoassays (Jiao et al., 2018).

**Figure 1:**
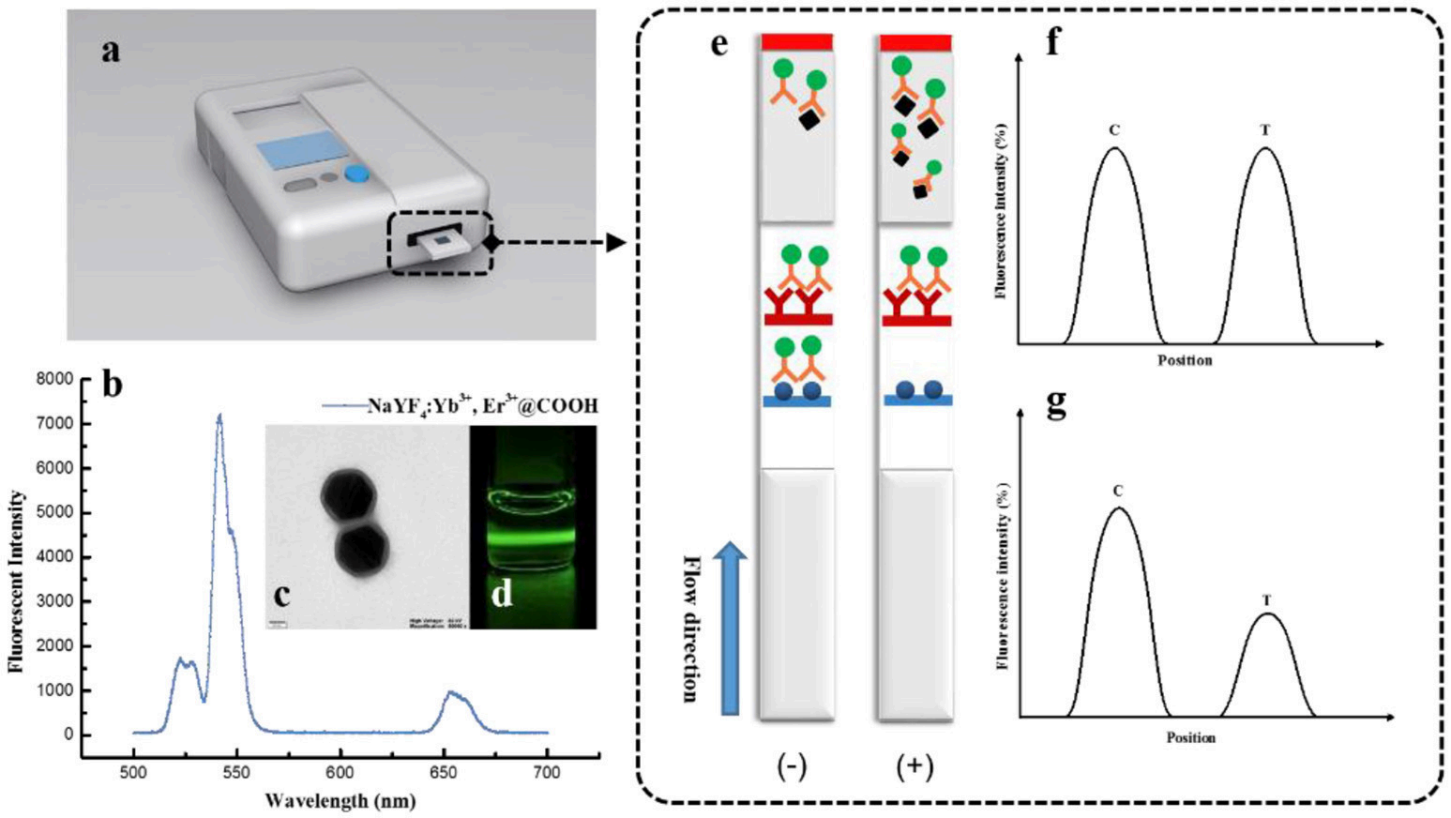
The detection principle of UCNP-LFIC strips. **(A)** Developed reader for UCNP-LFIC strips; **(B)** Emission spectra of carboxylic acid-functionalized UCNPs; **(C)** TEM image of carboxylic acid-functionalized UCNPs; **(D)** Emission of green light under 980 nm NIR excitation; **(E)** Diagram of test image; **(F)** Negative test result; **(G)** Positive test result.

The principle of UCNP-LFIC detection is presented in Figure 1. Firstly, UCNP-mAb probes were pre-mixed with analytes to form a uniform solution. Then the sample solution flowed simultaneously through the strip by capillary force. After reaction, the fluorescent signal intensity of strips was read by the portable machine, which integrated with the NIR laser system and translated the information into an electronic data-processing computer. There was competition between the immobilized antigen and target analyte for binding to the UCNP-mAb probe. The target analytes were able to combine with the probe specifically to form the UCNP-mAb-analyte complex, and the amount of UCNP-mAb probes captured by test line decreased, indicating a positive result (+) (Figure 1G). If the target analytes were absent in the sample solution or lower than the detectable ability of the assay, all UCNP-mAb probes would react with the test line. The excess particles flowed through the membrane continuously, and then became trapped with control line. This phenomenon resulted in a negative result (–) (Figure 1F).

### Development of the UCNP-LFIC Assay

The fluorescent intensity of nanoparticles is quite important in the assay. High fluorescent efficiency of single nanoparticles is beneficial for a more sensitive detection model (You et al., 2017). The dispersity and stability of UCNPs were measured via TEM and fluorescence spectrometer systems before conjugation. As a result, we observed that the size of the UCNPs was 140~150 nm, with a thickness of the SiO_2_ shell layer of ~10 nm (Figure 1C). With good dispersity, the UCNPs emitted green light under 980 nm NIR excitation for the doped of rare earth elements (Figures 1B,D). All characterizations indicated the UCNPs can be used for the development of fluorescent immunoassays.

Generally, crude UCNPs are a kind of inorganic material without active groups to combine with biomolecules. The surface modifications provided the ability to covalently conjugate with antibodies (Liang et al., 2017). In this work, the commercial UCNPs modified with carboxylic groups were coupled with mAb under the EDC/sulfo-NHS-mediated reaction. It should be noted that the quality of the UCNP-mAb probes is a key factor. The dispersion and size performance of the probes is directly relevant to the selection of nitrocellulose membranes. Differences in migration speed thus significantly affect the fluorescent intensity and the assay's sensitivity. Thus, the selection and optimization of an appropriate coupling system is crucial. The above conditions were optimized in this study (illustrated in part of Preparation of UCNPs-mAb probe). The freshly-prepared complex generally dispersed well in the preservation solution.

**Figure 2:**
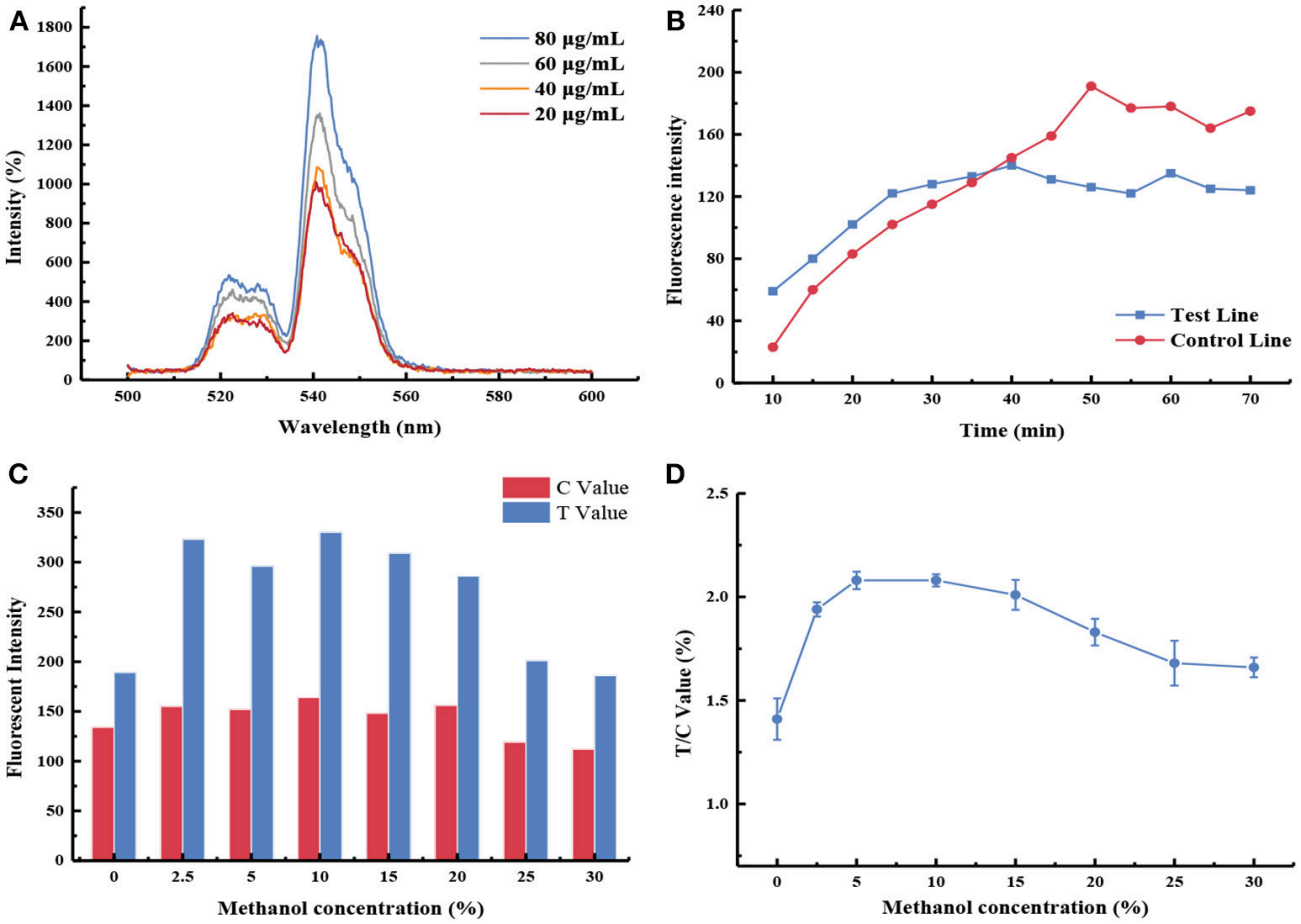
Optimization of UCNP-LF assay. **(A)** The fluorescence intensity emitted by different antibody labeling amounts; **(B)** Immunological kinetics analysis of probes on the strip; **(C)** The fluorescence intensity values of the test line (T Value) and control line (C Value) were recorded by using buffers containing different methanol concentrations; **(D)** Influence of methanol concentration in the FI_T_/ FI_C_ value.

After systematic optimization (Figure 2), 80 μg/mL of mAb was conjugated with UCNPs, and PA0304-OVA (3 mg/mL) and goat anti-mouse IgG (0.3 mg/mL) were immobilized on the test and control zones, respectively. PBS (0.01 M, pH 7.4) with a final methanol content of 10% was selected as the reaction buffer. Meanwhile, it was proved that 40 min was sufficient for strip reading (more details are available in the Supplementary Material).

### Evaluation of the UCNP-LFIC Assay

In a previous study, the produced mAb targeting parathion was identified through an indirect competitive chemiluminescence enzyme-linked immunoassay (CLEIA). It showed broad specificity to three OP pesticides, including parathion, methyl-parathion and fenitrothion, with IC_50_ values of 5.57, 2.30, and 2.62 ng/mL, respectively (Zou et al., 2017). The CLEIA indicated the mAb may have better affinity to two other analog compounds in this detection format.

In this work, we also selected parathion as a priority to test. Serial dilutions ranging from 0.39 to 500 ng/mL in the extraction solution were prepared and tested in quintuplicate, respectively.

**Figure 3:**
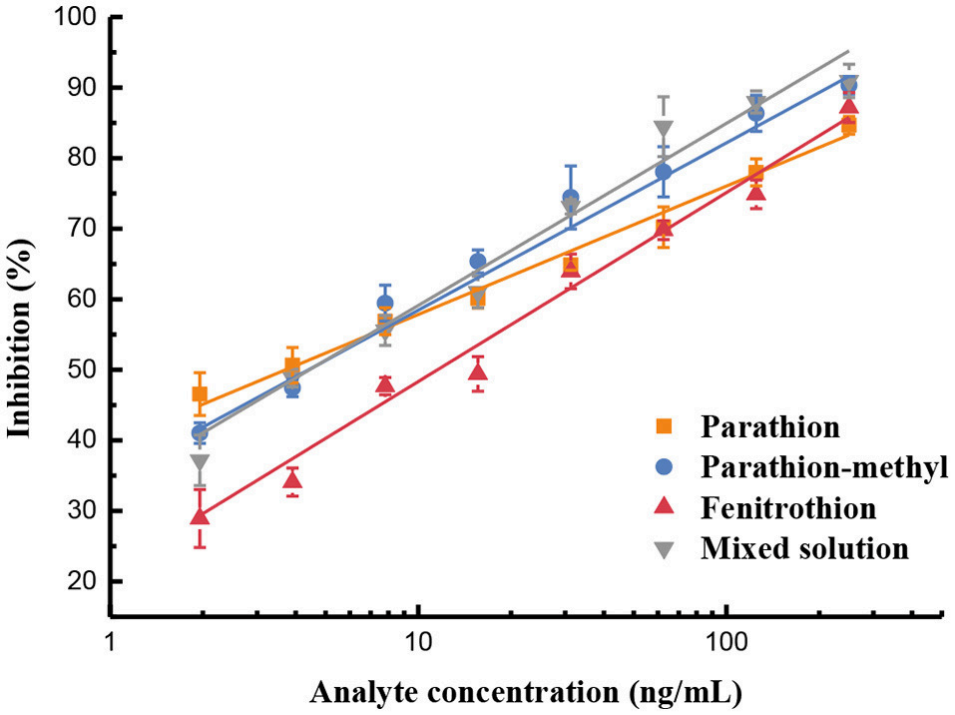
Sensitivity evaluation based on UCNP-LFIC of parathion, parathion-methyl, fenitrothion, and mixed solution.

Afterward, the standard curves for each and the mixed solution of the three OP pesticides were established (Figure 3). The indirect competitive UCNP-LFIC assay displayed good linear fitting (R^2^ ≥ 0.9776) within the analyte concentration ranging from 0.98/1.95 to 250 ng/mL (Table 1). According to China's food safety national standard allowance, the minimum concentration of linear detection range was lower than the maximum residue limit (MRL) value. The IC_50_ values of parathion, parathion-methyl and fenitrothion were 3.44, 3.98, 12.49 ng/mL, respectively. By comparison, there is quite different in the sensitivity order between CLEIA (homogeneous reaction mode) and the UCNP-LFIC assay (paper-based flow reaction mode) using the same mAb and competitive antigen. It indicates that the selection of the reaction mode has a significant influence on assay sensitivity. This conclusion was also demonstrated in the SPR-based immunoassay (Jiao et al., 2018).

**Table 1:** Linear regression equations based on UCNP-LFIC of OP pesticides (*n* = 5).

**Pesticide**	**Standard Curve**	***R*^**2**^**	**IC_**50**_ (ng/mL)**	**Linear range (ng/mL)**	**MRL**^**a**^ **(mg/kg)**
Parathion	y = 7.575 ln(x) + 40.64	0.9907	3.44	0.98~250	0.01
Parathion-methyl	y = 10.381 ln(x) + 35.67	0.9853	3.98	1.95~250	0.01
Fenitrothion	y = 11.702 ln(x) + 20.45	0.9851	12.49	0.98~250	0.1
Mixed solution	y = 11.517 ln(x) + 31.74	0.9776	4.88	1.95~250	–

a *MRL value refers to the maximum residue limit allowed in the national food safety standard method GB 2763-2016 of China*.

### Comparison Between the UCNP-LFIC and CG-LFIC Assays

Label selection is a vital factor in improving the sensitivity of LFIC. Two LFIC (CG-LFIC and UCNP-LFIC) assays were established based on the competitive format after systemic optimization. The CG-LFIC assay was established and optimized in accordance with the procedures reported in our lab (Guo et al., 2009). Selection of crucial working conditions was similar to the UCNP-LFIC assay, including the reaction buffer, the concentration of coating materials and methanol, and the amount of mAb labeled with CG nanoparticles.

**Figure 4:**
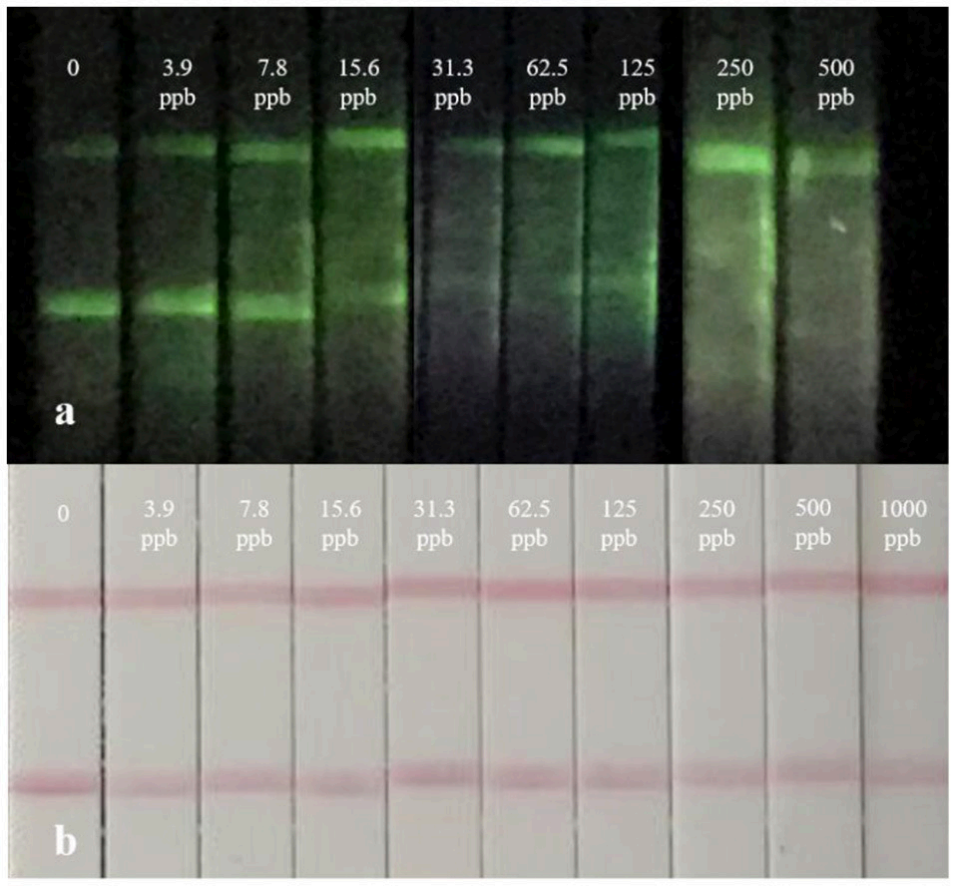
Real detection figure of UCNP-LFIC assay and GC-LFIC assay of mixed solution. **(A)** UCNP-LIFC assay. **(B)** GC-LFIC assay.

By adopting a series concentration of mixed standard solution, the sensitivity of strips was determined by the naked eye. The color of the test line in CG-LFIC assay remained even at an extreme high concentration of 1,000 ng/mL. It was too hard to meet the detection need when CG was applied as a label. Meanwhile, the same line nearly disappeared at a concentration of 15.6 ng/mL in the UCNP-LFIC assay (Figure 4). The results indicate that the UCNPs are more suitable than traditional CG as a label in this study. The variation is probably due to the properties of distinct labeling materials and different coupling methods. Furthermore, UCNPs have unique optical properties in size, color, and fluorescence intensity under different processes of synthesis and modification. The versatile performance thus enhances the potential for trace detection.

### Selectivity of UCNP-LFIC Assay

**Figure 5:**
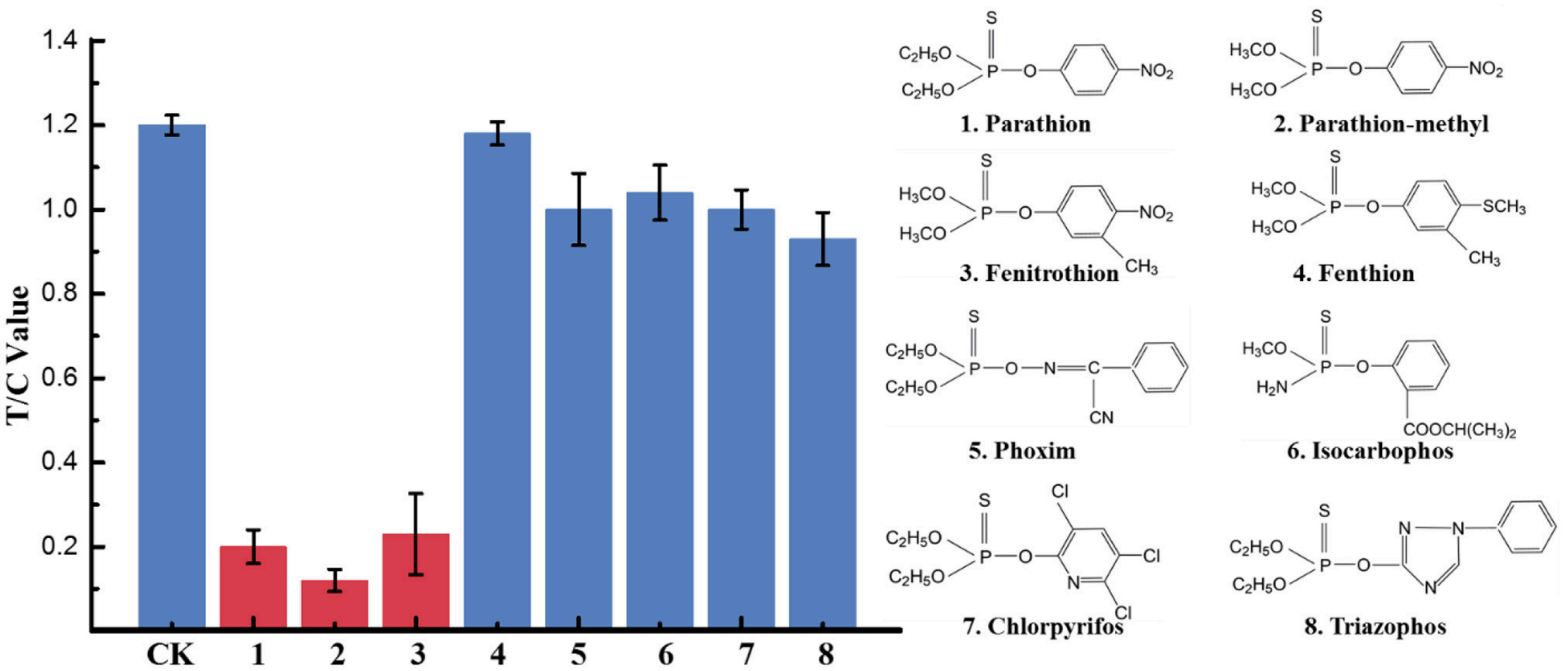
Selectivity evaluation of UCNP-LFIC. Eight OPs including parathion, parathion-methyl, fenitrothion, fenthion, phoxim, isocarbophos, chlopyrifos, and triazophos with a final concentration of 500 ng/mL.

To determine the specificity of the immunoassay, eight OP pesticides including parathion, parathion-methyl, fenitrothion, fenthion, phoxim, isocarbophos, chlopyrifos, and triazophos were investigated. Each analyte diluted to a standard solution with a final concentration of 500 ng/mL was tested by the developed UCNP-LFIC assay. The FI_T_/FI_C_ values of blank control and annalyte standard solutions were determined based on the corresponding test and control lines. In contrast, the inhibition rates by parathion, parathion-methyl, and fenitrothion were significantly higher than those from the other five compounds (Figure 5), suggesting the assay selectivity was consistent with the mAb specificity characterized in our previous work (Zou et al., 2017; Jiao et al., 2018).

### Matrix Interference in Real Samples

Immunoassay performance can be interfered with by the complicated matrix compositions in the samples, such as protein, lipid, pigment, and fiber. These components might cause antibody denaturation, non-specific absorption, and then affect the sensitivity.

**Figure 6:**
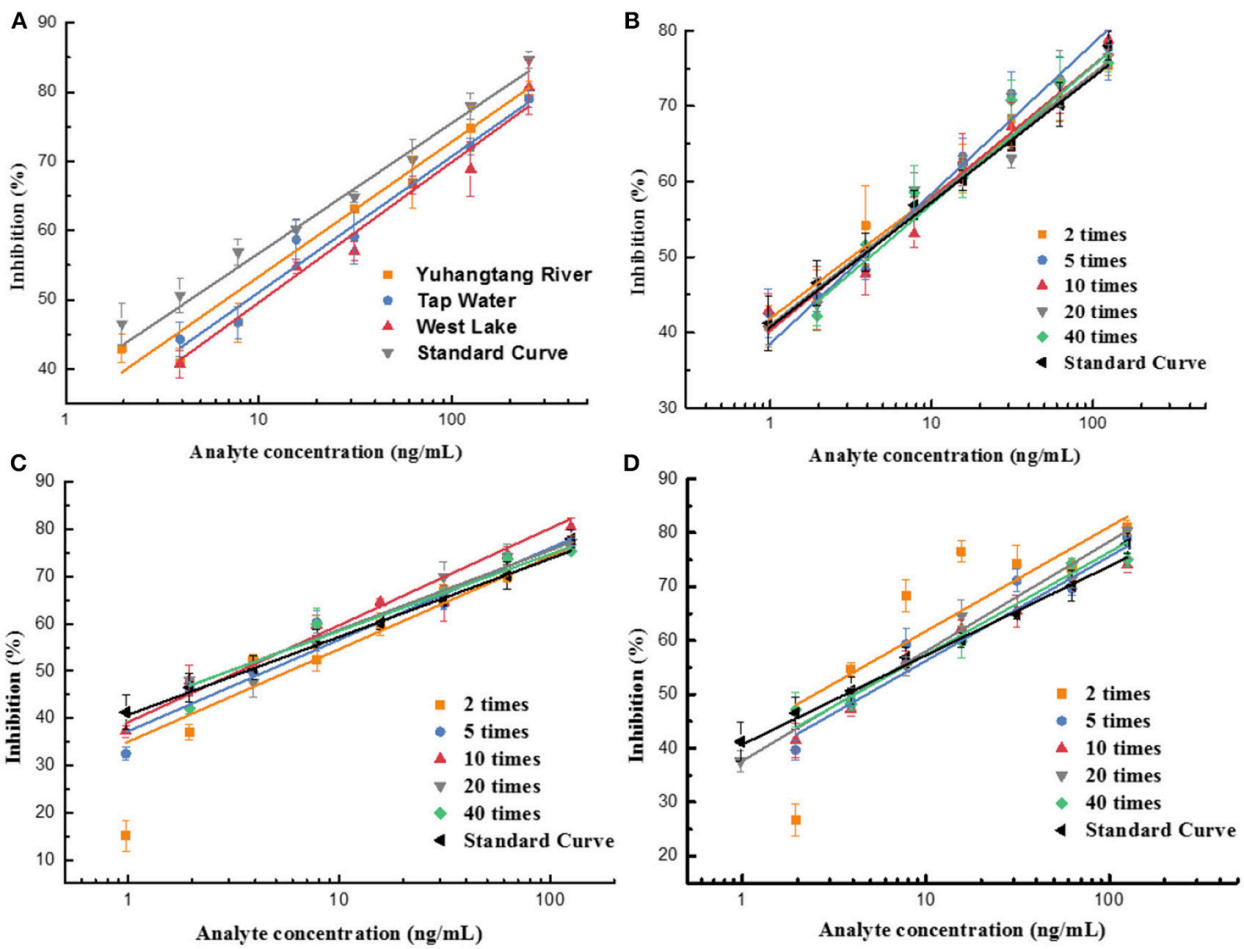
Fitted standard curve results of mixed OPs in different dilution times of real sample matrix. **(A)** Water. **(B)** Cucumber. **(C)** Tomato. **(D)** Orange.

In this study, the impact of the matrix in different vegetables, fruits, and water on the UCNP-LFIC assay's sensitivity was evaluated. To eliminate the matrix interference and guarantee the credibility of the method, sample dilution was conducted prior to detection. According to the pretreatment method, food sample extracts were diluted to 2, 5, 10, 20, and 40 times with PBS buffer containing 10% methanol. Then, a series of sample buffers with different matrix concentrations was used to prepare standard solutions. As shown in Table 2 and Figure 6, the matrix effect values of cucumber and tomato matrices were within 80–120% under 2 times' dilution. The results indicated the matrix interference could be ignored in the above dilution times (Matuszewski et al., 2003), whereas the orange matrix should be diluted 5 times, and there was no extra dilution for water.

**Table 2:** Matrix effect of food sample.

**Sample**	**Dilution**	**Standard curve**	***R*^**2**^**	**IC_**50**_ (ng/mL)**	**Matrix effect**
Cucumber	2	y = 7.2632 ln(x) + 41.578	0.9840	3.19	95.88%
	5	y = 8.003 ln(x) + 40.649	0.9765	3.22	105.65%
	10	y = 7.5116 ln(x) + 40.655	0.9710	3.47	99.16%
	20	y = 7.5349 ln(x) + 40.367	0.9790	3.59	99.47%
	40	y = 8.0456 ln(x) + 39.71	0.9604	3.59	106.21%
Tomato	2	y = 8.5744 ln(x) + 35.617	0.9524	5.35	113.19%
	5	y = 8.4773 ln(x) + 37.712	0.9528	4.26	111.91%
	10	y = 8.2276 ln(x) + 40.291	0.9698	3.25	108.61%
	20	y = 7.992 ln(x) + 40.146	0.9679	3.24	105.50%
	40	y = 7.9008 ln(x) + 39.713	0.9564	3.68	104.30%
Orange	2	y = 10.64 ln(x) + 35.738	0.7138	3.82	140.46%
	5	y = 8.855 ln(x) + 36.932	0.9483	4.37	116.90%
	10	y = 7.9104 ln(x) + 37.85	0.9731	4.65	104.43%
	20	y = 8.5783 ln(x) + 38.273	0.9880	3.92	113.24%
	40	y = 7.5503 ln(x) + 40.195	0.9705	3.66	99.67%

### Detection of Spiked Sample

In order to evaluate the accuracy of the UCNP-LFIC assay, we performed spiked recovery tests within the detection linear range of the assay. Various samples spiked with different concentrations of parathion and the mixed solutions of three OP pesticides (2.5, 5, and 10 ng/mL) were analyzed in triplicates. Based on the standard curve, the pesticide amount and recovery rate were calculated. At the same time, the precision and repeatability among batches were also evaluated by relative standard deviation (RSD). As listed in Table 3, the recovery rates ranged from 67 to 120% with RSD ≤ 19.54%, which generally satisfied the requirement of trace detection.

**Table 3:** Recovery rate determination of parathion and mixed solution of three OP pesticides by UCNP-LFIC strips.

**Sample**	**Spiked level (ng/g or ng/mL)**	**Parathion**			**Mixed solution**		
		**Detected amount** **(ng/g or ng/mL)**	**Recovery rate (%)**	**RSD (%)** **(*n =* 6)**	**Detected amount** **(ng/g or ng/mL)**	**Recovery rate (%)**	**RSD (%)** **(*n =* 6)**
Cucumber	5	5.78	116	2.20	3.60	72	3.53
	10	9.50	95	12.96	6.70	67	12.55
	20	19.48	97	7.49	21.50	108	8.39
Tomato	5	5.14	103	17.42	4.40	88	12.90
	10	9.04	90	15.77	7.28	73	4.71
	20	19.50	98	7.29	18.46	92	18.31
Orange	12.5	10.35	83	1.95	9.60	77	14.28
	25	28.90	116	27.90	26.25	105	19.54
	50	51.75	104	10.11	55.25	111	7.97
Yuhangtang River	2.5	2.05	82	14.20	1.97	79	9.36
	5	5.13	103	2.14	6.01	120	10.53
	10	9.48	95	10.32	10.22	102	11.98
Tap Water	2.5	1.87	75	12.42	2.81	112	4.31
	5	4.98	100	10.35	5.23	105	10.23
	10	11.29	113	11.94	9.21	92	15.23
West Lake	2.5	2.34	94	8.47	2.9	116	6.51
	5	5.02	100	10.62	4.56	91	9.12
	10	11.52	115	5.24	9.92	99	14.56

However, the UCNP-LFIC assay was not able to distinguish parathion, parathion-methyl, and fenitrothion, instead only indicating the mixture levels. In any case, it can work as a screening tool for large-scale multi-residue detection of the three OP pesticides and thus contribute to a reduction of the sample amount for further instrumental analysis.

## Conclusion

In this study, we successfully established a UCNP-based competitive LFIC strip for rapid multi-residue detection of three OP pesticides with good sensitivity in food samples. The UCNP-LFIC immunoassay showed a good linear fit (R^2^ ≥ 0.9776) in the range of 0.98/1.95–250 ng/mL, and high sensitivity for parathion, parathion-methyl, and fenitrothion (IC_50_ ranging from 3.44 to 12.49 ng/mL). Meanwhile, it improved the determination of multiple OP pesticide residues from qualitative to semi-quantitative detection within 40 min. This is the first report on using a UCNP-LFIC assay for OP pesticide residue detection. The assay revealed the broad prospects of UCNPs for the detection of pesticides and other small contaminant molecules.

However, the technical process for UCNP-LFIC strips should be further improved. Although the fresh synthesized UCNP-mAb probes have homogeneous dispersibility, the stock solution of UCNP-mAb probes was aggregated to form flocculent precipitates after 2 days' storage. Careful optimization of the probes' preparation method and the preservation buffer may solve this problem to some extent. In view of high requirements in the analytical field, it is still necessary to improve UCNP-LFIC sensitivity for trace detection. The main approaches include the synthesis of new UCNPs with high luminous intensity, the modification of UCNPs' surface with more active sites, and the development of a UCNP detector apparatus with enhanced optical performance.

## Ethics Statement

This study on mice was carried out in accordance with the recommendations of the animal welfare committee of Zhejiang University in China.

## Author Contributions

YG and YihL designed the study and guided the experiments. RZ carried out experiments, analyzed data, and wrote the manuscript draft. YC, TZ, and FS assisted with experiments. YinL and YZ assisted with data analysis. MZ, XY, XQ, and GZ helped to design the study and supply the facility. YG and YihL revised the manuscript.

### Conflict of Interest Statement

The authors declare that the research was conducted in the absence of any commercial or financial relationships that could be construed as a potential conflict of interest.
