# Supplementary material for: Up-Converting Nanoparticle-Based Immunochromatographic Strip for Multi-Residue Detection of Three Organophosphorus Pesticides in Food
Source: Front Chem. 2019 Feb 7;7:18. doi: 10.3389/fchem.2019.00018 (PMC6374334; doi:10.3389/fchem.2019.00018)
Supplement: Supplementary file 1 [file Data_Sheet_1.docx]

**Supplementary Material**

**Article:** Up-converting nanoparticles-based immunochromatographic strip for multi-residue detection of three organophosphorus pesticides in food

Rubing Zou^a^, Yunyun Chang^a^, Tianyi Zhang^a^, Fangfang Si^a^, Ying Liu^a^, Ying Zhao^a^, Yihua Liu^a,b^ *, Mingzhou Zhang^c^, Xiaoping Yu^c^, Xusheng Qiao^d^, Guonian Zhu^a^, Yirong Guo^a^*

^a^ Institute of Pesticide and Environmental Toxicology, Ministry of Agriculture Key Laboratory of Molecular Biology of Crop Pathogens and Insects, Zhejiang University, Hangzhou 310058, China

^b^ Research Institute of Subtropical Forestry, Chinese Academy of Forestry, Hangzhou

311400, China

^c^ College of Life Sciences, China Jiliang University, Hangzhou, 310018, China

^d^ Department of Materials Science and Engineering, Zhejiang University, Hangzhou 310027, China

*The corresponding authors, E-mail: [yirongguo@zju.edu.cn](mailto:yirongguo@zju.edu.cn); [liuyihua@zju.edu.cn](mailto:liuyihua@zju.edu.cn).

**1 Optimization of UCNPs-LFIC assay parameters**

**1.1 Working concentration of immuno-reagents**

Different concentrations of antibody (20, 40, 60 and 80 μg/mL) were prepared. Fluorescence intensity and sensitivity were compared under different immobilized concentration in the test and control lines, respectively.

As shown in Figure 2a, the fluorescence intensity of the probe increased with the antibody within proper concentration. It was speculated that the interesting result might be caused by various weight of complex possessing different centrifugal elution. To ensure the fluorescence intensity, 80 μg/mL of mAb was labeled with UCNPs to prepare probes.

Based on the checkerboard titration method, the amount of biological reagents on the test strips and the antibody coupling to the probes were adjusted. Then, standard curves were established respectively. After systematic optimization, PA0304-OVA (3 mg/mL) and goat anti-mouse IgG (0.3 mg/mL) were immobilized on the test and control lines, respectively.

**1.2 Reaction time**

As a paper phase model, the migration of the fluorescent probes in the liquid depends on capillary force. In addition, the material amount of the test region is limited. The signal value will tend to stabilize after a period of reaction. Meanwhile, the size and homogeneity of probes in solution are important. With small-sized and good dispersity, the detection objects exhibit a faster migration rate which has an impact on the immunological reaction on the strips ^[1]^. Hence, sufficient reaction time is necessary.

According to the color indication of the pH test paper, it needs at least 15 mins’ incubation for the fluid upwelled to endpoint. In the immunological kinetics analysis, the fluorescent intensity of test line (FI_T_) and control line (FI_C_) were recorded every 5 min in 1 h. The kinetics curve was fitted by plotting both FI_T_ and FI_C_ against time. As demonstrated in Figure 2, the FI_C_ is correlated with time positively, presenting the continuous increase trend over preset time. However, FI_T_ tends to maintain balance and the FI_T_/ FI_C_ value is close to 1:1 in approximately 40 min. Thus, 40 min was sufficient for immunological reaction.

**1.3 Concentration of methanol in working buffer**

In previous study, a certain volume of organic solvent in buffer favored the sensitivity has been proved ^[2]^. However, the immunoreaction between antigen and antibody was disturbed accessibly under high concentration of organic solvent. To evaluate the effects of methanol contents on the assay performance, we diluted the pesticide standard solution with PBS buffer (0.01 mol/L, pH 7.4) containing different volumes of methanol. The final concentration of methanol in the reaction buffer was from 0% to 30% (v/v). Figure 2 demonstrates that FI_C_ tends to be stable with the increase of methanol concentration. We speculated that goat anti-mouse IgG can tolerate the series of organic solvent concentration. However, FI_T_ showed a rapid increase from 0% to 2.5%, and then kept a balance at a concentration between 2.5% and 20%. Finally, the signal tends to decrease gradually with the concentration over 20% to 30%. FI_T_/ FI_C_ value shows a nearly same trend and reached a peak at 5% (Figure 2). Thus, we selected PBS buffer (0.01 mol/L, pH 7.4) with final methanol concentration of 10% as dilute and extract solution for real sample.

**Supplementary References**

[1] Duan, H., Huang, X. L., Shao, Y. N., Zheng, L. Y., Guo, L., Xiong, Y. H. (2017). Size-dependent Immunochromatographic Assay with Quantum Dot Nanobeads for Sensitive and Quantitative Detection of Ochratoxin A in Corn. Analytical Chemistry 89: 7062-7068.

[2] Taheri, N., Lan, M. J., Wei, P., Liu, R., Gui, W. J., Guo, Y. R., et al. (2016). Chemiluminescent Enzyme Immunoassay for Rapid Detection of Three α-Cyano Pyrethroid Residues in Agricultural Products. Food Analytical Methods 9, 1-10. doi: 10.1007/s12161-016-0482-x

**Supplementary Figures**


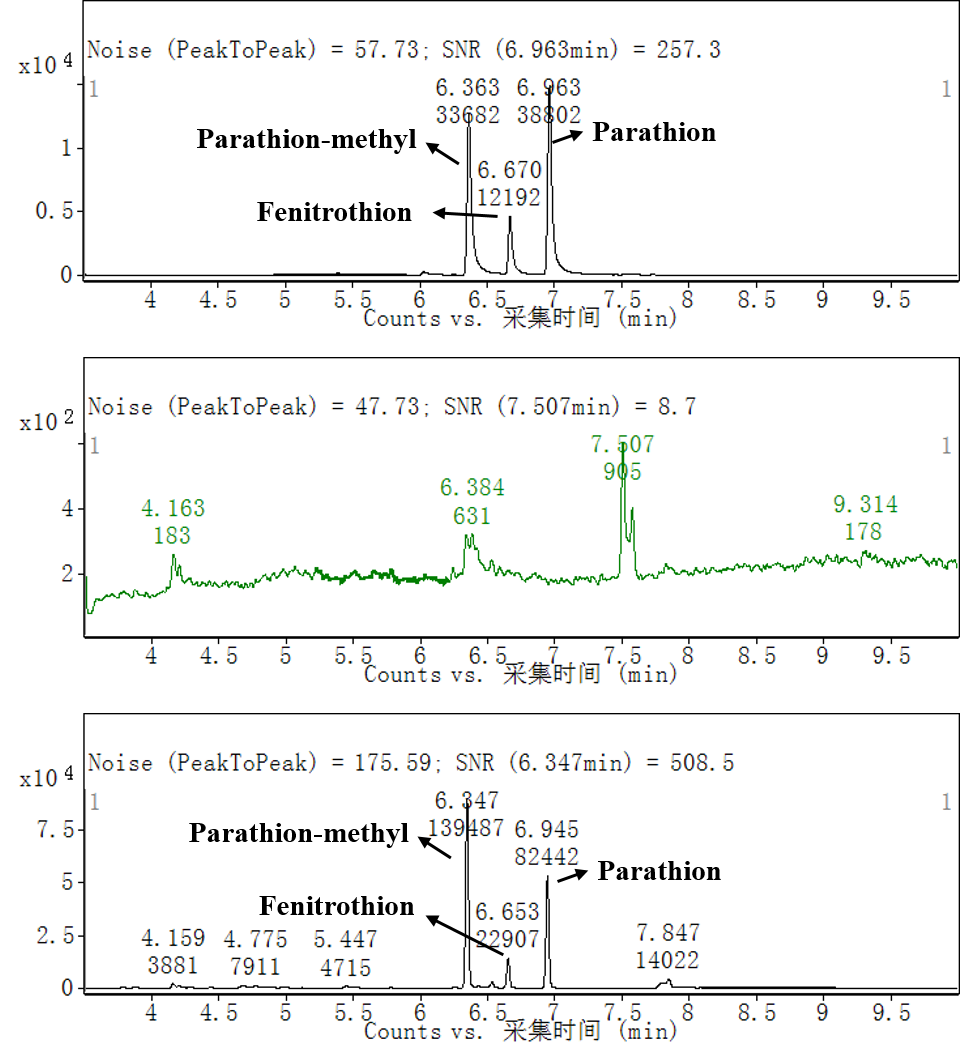


**Figure S1. Sample detection in GC-MS (**a. Standard solution; b. OPs-free orange sample verification before recovery test; c. Spiked orange.**)**
